# Relationship between grip strength and newly diagnosed nonalcoholic fatty liver disease in a large-scale adult population

**DOI:** 10.1038/srep33255

**Published:** 2016-09-12

**Authors:** Ge Meng, Hongmei Wu, Liyun Fang, Chunlei Li, Fei Yu, Qing Zhang, Li Liu, Huanmin Du, Hongbin Shi, Yang Xia, Xiaoyan Guo, Xing Liu, Xue Bao, Qian Su, Yeqing Gu, Huijun Yang, Bin Yu, Yuntang Wu, Zhong Sun, Kaijun Niu

**Affiliations:** 1 Nutritional Epidemiology Institute and School of Public Health, Tianjin Medical University, Tianjin, China; 2 Collaborative Innovation Center of Non-communicable Disease, Tianjin Medical University, Tianjin, China; 3 Health Management Centre, Tianjin Medical University General Hospital, Tianjin, China

## Abstract

Enhanced muscle strength is often related to improved insulin sensitivity and secretion, control of lipid metabolism, and increased secretion of myokines. These factors have emerged as important mechanisms involved in the development and progression of nonalcoholic fatty liver disease (NAFLD), implying that muscle strength may be a useful predictor for NAFLD. We aimed to assess the relationship between grip strength (GS) and NAFLD in a large-scale adult population. GS was assessed using an electronic hand-grip dynamometer, and NAFLD was diagnosed by the liver ultrasonography. Multiple logistic regression analysis was used to assess the relationship between the quartiles of GS per body weight and the prevalence of NAFLD. After adjusting for potentially confounding factors, the odds ratios (95% confidence interval) for overall NAFLD, NAFLD with normal alanine aminotransferase levels, and NAFLD with elevated alanine aminotransferase levels across the quartiles of GS were 1.00 (reference), 0.89 (0.78, 1.01), 0.77 (0.67, 0.89), and 0.67 (0.57, 0.79); 1.00 (reference), 0.91 (0.80, 1.04), 0.79 (0.68, 0.92), and 0.72 (0.61, 0.85); 1.00 (reference), 0.77 (0.61, 0.98), 0.67 (0.51, 0.86), and 0.53 (0.40, 0.71) (all *P* for trend < 0.01), respectively. This is the first study shows that increased GS is independently associated with lower prevalence of NAFLD.

Nonalcoholic fatty liver disease (NAFLD), characterized by an abnormal accumulation of fat in hepatocytes, is currently the most common cause of chronic liver disease in industrialized countries^1^,^2^. NAFLD is emerging as a leading cause of chronic liver disease and people with NAFLD are at increased risk for cardiovascular disease (CVD) and liver-related mortality^3^,^4^. NAFLD is a major health burden, affecting 5–28% of adults in Asia–Pacific countries^5^. With the westernization of lifestyles, the prevalence of NAFLD in developing countries has increased rapidly in last decade due to the pandemic of diabetes and obesity^6^.

Accumulated evidence has shown that muscle-strengthening activities are closely related to improved insulin sensitivity, modulated insulin secretion and ameliorated dyslipidaemias^7^,^8^,^9^,^10^, which are the principal risk factors for developing NAFLD^11^,^12^. Skeletal muscle, a major site of insulin- and exercise-stimulated glucose disposal, might interact with the pancreas. Working skeletal muscle produces a variety of biologically and metabolically bioactive factors, collectively called myokines, such as myostatin and interleukin-6 (IL-6) to participate in the regulation of insulin secretion and insulin resistance^7^,^8^. Moreover, myonectin and irisin are recently discovered myokines produced and secreted by skeletal muscle, which appear to be involved in the regulation of insulin resistance and lipid metabolism^9^,^10^. Because these factors have emerged as important mechanisms involved in the development and progression of NAFLD, we speculated that muscle strength may be an important predictive factor or therapeutic target for NAFLD.

Currently, three cross-sectional studies have focused on the relationships between skeletal muscle mass and NAFLD in the Korean population^13^,^14^,^15^. However, several large-scale cohort studies have identified that muscle strength decline is much more rapid than muscle mass, maintaining or gaining muscle mass does not prevent aging-related declines in muscle strength, and low muscle mass does not explain the strong association of muscle strength with mortality^16^,^17^. Therefore, it is conceivable that the assessment of muscle strength could be a more valid and valuable value than muscle mass when assessing how to prevent and treat NAFLD in its very early stages and how to decrease its long-term severity. On the other hand, grip strength, which was selected as the primary measure of muscle strength in this study, is easy to use in both clinical and community settings^18^,^19^. Therefore, we supposed that GS could be a feasible and cost effective indicator when screening for NAFLD especially in developing countries. Moreover, a large number of epidemiological studies have consistently reported that GS in healthy adults is a reliable risk-stratifying method for functional limitations and disability for more elderly adults as well as for all-cause death, and CVD^17^,^18^,^19^,^20^. However, to best of our knowledge, no previous studies have reported the relationship between GS and NAFLD. Thus, we performed an adult population-based study to investigate the relationships between GS and new-diagnosed NAFLD.

## Results

In this study, 51.0% (10,679 of 20,957) for participants were males and 49.0% (10,278 of 20,957) were females, with mean ages (standard deviation) of 41.2 (12.0) years. The overall prevalence of NAFLD was 27.0% (39.0% and 14.5% for males and females respectively).

### Characteristic of participants

Characteristics of participants according to the quartiles of GS per body weight are presented in Table 1. Compared with participants in the lowest quartile of GS per body weight, participants in the upper three quartiles tended to be younger and to have higher serum alanine aminotransferase (ALT) concentrations, and physical activity (PA) and lower body mass index (BMI). A higher proportion of these participants were males, with a higher proportion of current smokers, ex-smokers, alcohol consumers, educational levels, professionals, and household income and a lower proportion of metabolic syndromes (MS) diabetes, hyperlipidemia and family history of CVD, hypertension and diabetes (*P* for all trends < 0.01). Otherwise, no significant difference was observed between quartiles of GS per body weight.

### Grip strength and NAFLD

The crude and adjusted relationships between quartiles of GS per body weight and NAFLD is presented in Table 2. In the final multivariate model, the odds ratios (OR) (95% confidence interval (CI)) for overall NAFLD, NAFLD with normal ALT levels, and NAFLD with elevated ALT levels across the quartiles of GS were 1.00 (reference), 0.89 (0.78, 1.01), 0.77 (0.67, 0.89), and 0.67 (0.57, 0.79); 1.00 (reference), 0.91 (0.80, 1.04), 0.79 (0.68, 0.92), and 0.72 (0.61, 0.85); 1.00 (reference), 0.77 (0.61, 0.98), 0.67 (0.51, 0.86), and 0.53 (0.40, 0.71) (all *P* for trend < 0.01), respectively. Similar relationships were also observed when sex-stratified analyses were performed to investigate the association between GS and NAFLD. The adjusted OR (95% CI) of NAFLD for increasing quartiles of GS per body weight were 1.00 (reference), 0.84 (0.73, 0.95), 0.83 (0.72, 0.95), and 0.57 (0.49, 0.67) (*P* for trend < 0.001) in males and 1.00 (reference), 1.09 (0.92, 1.30), 0.85 (0.69, 0.95), and 0.84 (0.64, 0.96) (*P* for trend < 0.001) in females. No significant interaction of GS per body weight with other confounders for NAFLD in the final models was observed (all *P* for interactions > 0.18). We further performed a sensitivity analysis after excluding the subjects who reported taking anti-hyperlipidemia drugs (n = 786). However, the relationship between GS and NAFLD did not change. The adjusted OR (95% CI) of NAFLD for increasing quartiles of GS per body weight were 1.00 (reference), 0.91 (0.80, 1.03), 0.78 (0.67, 0.90), and 0.68 (0.58, 0.80) (*P* for trend < 0.001). The crude risk difference for identifying one patient with NAFLD for each quartile was 3.3%, 1.2%, and 8.8%, so the number needed to evaluate for GS in order to identify one additional patient likely to have NAFLD was 30, 84, and 12 for each quartile.

## Discussion

This is the first study to assess the relationship between GS per body weight and newly diagnosed NAFLD in such a large general population. The results demonstrated that subjects with lower GS per body weight had a higher prevalence of NAFLD after adjusted for confounding factors.

NAFLD has become the most common chronic liver disease in many Asia Pacific countries, including China. Based on surveys using ultrasonography, the prevalence of NAFLD in the general population across Asia varies from 5% to 40%, and for Chinese populations, varies from 5% to 24%, being higher in urban areas than rural ones^5^. Furthermore, a recent meta-analysis of 48 studies demonstrated that the overall pooled prevalence of NAFLD was 20.1% and that NAFLD prevalence continues to increase with age in the mainland of China^21^. In our study population, the overall prevalence of NAFLD was 27.9%, which is higher than previously reported^5^,^21^. Because age and living environment were closely related to the prevalence of NAFLD, we considered that higher age structure (mean age: 41.2y) and the gradually westernization of lifestyles in urban population are main reasons for the higher prevalence of NAFLD in the present study.

Three Korean’s cross-sectional studies have assessed the relationship between muscle mass and NAFLD^13^,^14^,^22^. These studies consistently indicated that individuals with lower muscle mass have a higher risk of NAFLD compared with individuals with a preserved muscle mass. However, muscle mass, as a static index, often tends to lag behind muscle function. A well-designed and executed large-scale longitudinal study demonstrated that muscle strength decline is much more rapid than the concomitant loss of muscle mass, and maintaining or gaining muscle mass does not prevent age-related declines in muscle strength^16^. Another cohort study have clearly indicated that low muscle mass did not explain the strong association of strength with mortality, suggesting that muscle strength as a marker of muscle quality is more important than quantity in estimating mortality risk^17^. Therefore, the assessment of muscle strength may be more valuable for prediction or treatment in the very early stages of NAFLD than muscle mass. On the other hand, GS is a simple, non-invasive marker of muscle strength of upper extremities and correlates well with other muscle function tests such as knee extension strength or peak expiratory flow^23^. Consequently, GS was selected to assess the relationship between muscle strength and NAFLD in this study. In the present study, the results showed that the prevalence of NAFLD was 33% lower for participants in the highest quartile than for those in the lowest quartile of GS.

We found that the significant relationship between NAFLD and GS remained even after controlling for comorbid conditions, such as MS, physical inactivity, diabetes, hyperlipidemia, and family history of diseases in the study population. This finding suggests that muscle weakness in NAFLD cannot be explained solely by comorbidity status, nutrition or physical inactivity but, rather, may have a specific pathophysiological pathway. The pathophysiological mechanisms between GS and NAFLD are multifactorial, but still not fully understood. Skeletal muscle, as a target organ for insulin action, plays an important role in insulin sensitivity and insulin resistance, which are known as key factors in the pathophysiology for NAFLD^8^. It can secrete multiple active factors (myokines). Myostatin and IL-6 are well-known skeletal muscle-secreted protein. Myostatin, a negative regulator of muscle growth, has recently been shown to play an important role in metabolism^24^. Myostatin expression is increased in dyslipidaemia by inhibiting brown adipocyte differentiation^24^. The increased myostatin also is likely to be a potent inducer of insulin resistance^25^. In turn, IL-6 promotes myogenic differentiation of skeletal muscle cells^26^. Accumulated evidences have showed IL-6 enhances insulin secretion either in cells or in humans^27^,^28^. Furthermore, myonectin^29^ and irisin^30^, as important myokins secreted by skeletal muscle, were discovered very recently. Myonectin and irisin are likely to be involved in lipid and glucose metabolism and thus may prevent the development of dyslipidemias and insulin resistance^9^,^29^,^30^. Therefore, it is plausible that muscle could play a causative role for NAFLD by secreting various myokines.

This present study had several advantages over previous studies. Firstly, this is the first study to assess the relationship between muscle functions and newly diagnosed NAFLD directly in a large-scale adult population. Moreover, measurement of GS is simple, inexpensive and non-invasive^19^. Finally, this was a recent, large population-based analysis using well-examined data, which strengthens the statistical reliability of the results. We adjusted for other potential confounding factors as much as possible, such as socio-demographic variables, lifestyle factors, socioeconomic status and history of diseases. Additionally, we excluded participants if they had a previous diagnosis of NAFLD, which may affect lifestyle changes that can confuse the actual relationship between GS and NAFLD.

To appreciate these findings, several aspects have to be taken into consideration. Firstly, liver biopsy, the gold standard in the diagnosis of liver disease, was not available in the present study, due to the apparently healthy study population. Instead, we used hepatic ultrasonography scanning to detect fatty liver disease. This technique has a sensitivity of 89% and a specificity of 93%^31^ and is wildly used in population-based studies due to its noninvasiveness and easy accessibility^5^. Another possible limitation is that we cannot assess a causal relation because of the cross-sec tional study design. Further prospective studies and intervention trials should be undertake to establish a causal relationship between GS and NAFLD. Moreover, we did not measure skeletal muscle fiber type conclusively. Thus we were unable to ascertain whether the negative relationship between GS and NAFLD is mediated by changes in muscle fiber size or fiber type distribution.

In conclusion, this is the first study shows that GS is inversely associated with newly diagnosed NAFLD. Further large prospective epidemiologic studies are needed to investigate the impact of skeletal muscle strength on the incidence of NAFLD in the future.

## Methods

This cross-sectional study is part of the Tianjin Chronic Low-grade Systemic Inflammation and Health (TCLSIHealth) Cohort Study, which is a large prospective dynamic cohort study committing on the relationships between chronic low-grade systemic inflammation and the health status of a population living in Tianjin, China^32^,^33^. Participants, who had received health examinations, including medical examinations, such as blood tests, abdominal ultrasonography, etc., and had completed questionnaires regarding their smoking and drinking habits and disease history over the course of January 2007 to December 2015, were recruited. Moreover, a detailed lifestyle questionnaire covering family income, marital status, employment status, educational level, PA, dietary habits, and use of medicines as well as physical performance tests were administered to randomly selected subjects from this population since May 2013. Ethical approval was given by the medical ethics committee of Institutional Review Board of the Tianjin Medical University with the reference number of TMUhMEC 201430. The methods of this study were carried out in accordance with the approved guidelines. All subjects provided written informed consent before enrolment.

We used the baseline data of the TCLSIHealth cohort study from 2013 to 2015. During the research period there were 25,060 participants who had received comprehensive health examinations including GS test and a comprehensive lifestyle questionnaire. We excluded participants who did not complete data collection on ALT (n = 107) or those with a history of CVD (n = 1,690) or cancer (n = 379). We also excluded participants with liver diseases (history of NAFLD, chronic hepatitis B or C, operation on liver, autoimmune liver diseases, cirrhotic or liver cancer) (n = 79) or AFLD (n = 1,848). Owing to these exclusions, the final study population comprised 20,957 participants (10,679 men and 10,278 women). For analysis of participants with NAFLD with normal ALT levels, we excluded participants with NAFLD with elevated ALT levels (n = 1,332). When assessing relationship between NAFLD with elevated ALT levels and GS, we excluded participants who had NAFLD with normal ALT levels (n = 4,327).

### Definition of NAFLD

Fatty liver disease (FLD) was diagnosed by real-time ultrasonography using standardized criteria performed by experienced technicians^34^. Positive abdominal ultrasound images contained: diffusely increased liver near field ultrasound echo (‘bright liver’) and increased liver echo texture when compared to the kidneys; vascular blurring and the gradual attenuation of far field ultrasound echo. Diagnosis of FLD required at least two of the abnormal findings listed above^35^. Participants with sonographic FLD and a self-reported weekly alcohol intake of <140 g and <70 g for males and females, respectively, were classified as having NAFLD^36^. ALT levels >41 U/L and >33 U/L for males and females, respectively, were considered to have NAFLD with elevated ALT levels.

### Assessment of GS

Voluntary isometric muscle strength (measured in kg) was measured using an electronic hand-grip dynamometer (EH101; CAMRY, Guangdong, China). Participants were tested by the trained technicians under the same conditions. Dynamometer width was adjusted for optimal fit for each participant according to instructions on the dynamometer^37^. Before the measurement, the dynamometers were calibrated using a back-loading rig and were within 0.1 kg error range. The evaluator gave verbal encouragement to elicit maximal performance from the participants during measurement. Forces were measured twice for each hand, and the greatest force was used for the analyses. Grip strength relative to body weight (kg/kg) also was calculated because of involvement of body weight in the maximal performance of muscle strength^38^,^39^,^40^. The test variability for maximal voluntary force on the part of the same subject was below 5%. Participants gave written informed consent prior to participation in the study. Ethical approval was given by the medical ethics committee of Institutional Review Board of the Tianjin Medical University.

### Assessment of alcohol consumption and total energy intake

Dietary habits was collected using a validated self-administered food frequency questionnaire that included 98 food items, with specified serving sizes described in terms of the natural portion or the standard weight and volume measurement of servings commonly consumed in general Chinese populations^32^,^41^,^42^. The FFQ included 8 frequency categories ranging from ‘almost never drink’ to ‘(≥4 servings per day’ for beverages. The mean daily intake of nutrients was calculated by using an ad hoc computer program developed to analyze the questionnaire. The Chinese food composition tables^43^ were used as the nutrient database. The reproducibility and validity of the questionnaire were assessed in a random sample of 150 participants and living in Tianjin by comparing the data from the questionnaire with the data from 2 dietary questionnaires collected approximately 3 months apart and 4-day weighed dietary records (WDRs). Spearman’s rank correlation coefficient for energy intake by the WDRs and the FFQ was 0.49. Correlation coefficients for alcohol (high-alcohol liquor, low-alcohol liquor, beer, white wine, red wine, and other alcoholic beverages) by the WDRs and the FFQ ranged from 0.32 to 0.58.

### Assessment of other variables

Blood samples for analysis of fasting blood glucose (FBG) and lipids were collected in siliconized vacuum plastic tubes. FBG was measured using the glucose oxidase method, total cholesterol (TC) and triglycerides (TG) were measured by enzymatic methods, low density lipoprotein (LDL) cholesterol was measured by the polyvinyl sulfuric acid precipitation method, high-density lipoprotein (HDL) cholesterol was measured by chemical precipitation, ALT was measured by International Federation of Clinical Chemistry method using reagents from Roche Diagnostics on an automatic biochemistry analyzer (Roche Cobas 8000 modular analyzer, Mannheim, Germany). Blood pressure (BP) was recorded as the mean of two measurements taken from the upper left arm at the brachial artery using an automatic device (Andon, Tianjin, China) after 5 minutes of rest in a seated position.

Metabolic syndrome was defined in accordance with the criteria of the American Heart Association scientific statements of 2009^44^. Diabetes was defined in accordance with the criteria of the world health organization^45^. Participants were considered to have diabetes when their FBG accorded with level of ≥7 mmol/L or physician-diagnosed diabetes and/or current use of antidiabetic medications. Participants were considered to have hyperlipidaemia when their total cholesterol ≥ 5.17 mmol/L or triglycerides ≥1.7 mmol/L or low density lipoprotein cholesterol ≥3.37 mmol/L or history of hyperlipidaemia^46^.

Anthropometric parameters (height and body weight) were recorded using a standard protocol. BMI was calculated as weight/height^2^ (kg/m^2^). The sociodemographic variables, educational level, employment status, smoking status and drinking status were obtained from a questionnaire survey.

Physical activity in the most recent week was assessed using the short form of the International Physical Activity Questionnaire (IPAQ)^47^. The questionnaire asked whether subjects had performed any activities from the following categories during the previous week: walking; moderate activity (household activity or child care); vigorous activity (running, swimming, or other sports activities). Metabolic equivalent (MET) hours per week were calculated using corresponding MET coefficients (3.3, 4.0 and 8.0, respectively) according to the following formula: MET coefficient of activity × duration (hours) × frequency (days). Total PA levels were assessed by combining separate scores for different activities.

### Statistical analysis

The descriptive data are presented as the mean (95% confidence interval, CI) for continuous variables, and as percentages for categorical variables. In order to characteristics of participants according to the quartiles of GS per body weight, continuous variable were examined using analysis of variance and logistic regression analysis for categorical variables. NAFLD was used as dependent variables, and GS per body weight categories in quartiles were used as independent variables. Relationship between GS and NAFLD status were examined using logistic regression by three different models. OR and 95% CI were calculated as well. Model 1 was used to calculate the crude OR, and model 2 was adjusted for age, sex and BMI. Model 3 additionally adjusted for MS, smoking status, drinking status, PA, total energy intake, educational levels, employment status, household incomes, diabetes, hyperlipidemia and family history of diseases including CVD, hypertension, hyperlipidemia, and diabetes. A linear trend across increasing quartiles was tested by using the median value of each quartile as an ordinal variable. Moreover, considering that sex is the most dominant factor impacting GS, the interaction between quartiles of GS and sex was tested by the addition of this cross-product term to the regression model. The interactions between GS per body weight and other confounders for having NAFLD were also tested. Furthermore, because statin use are common in hyperlipidemia patients and may cause a myopathy^48^. Therefore, a sensitivity analysis was performed after excluding the subjects who reported taking anti-hyperlipidemia drugs. Finally, we also calculated the crude risk difference for identifying one patient with NAFLD to determine the number needed to access (the same as the number needed to treat (NNT)) to identify one additional patient likely to have NAFLD for each quartile. The NNT is defined as the number of persons needed to treat to prevent one outcome, and is calculated by the inverse of the absolute risk reduction^49^. All statistical analyses were performed using the Statistical Analysis System 9.3 edition for Windows (SAS Institute Inc., Cary, NC, USA). All tests were two-tailed and *P* < 0.05 was defined as statistically significant.

## Additional Information

**How to cite this article**: Meng, G. *et al*. Relationship between grip strength and newly diagnosed nonalcoholic fatty liver disease in a large-scale adult population. *Sci. Rep.* **6**, 33255; doi: 10.1038/srep33255 (2016).

## Figures and Tables

**Table 1:** Participant Characteristics by Quartiles of Grip Strength per Body Weight^a^.

Characteristics	Quartiles of grip strength per body weight (range, kg/kg)						*P* for trend^b^
	Level 1 (0.08–0.40)	Level 2 (0.40–0.48)	Level 3 (0.48–0.56)	Level 4 (0.56–1.12)			
No. of subjects	5,240	5,239	5,238	5,240	
Age (y)	42.1 (41.7, 42.1)^c^	39.3 (38.9, 39.3)	39.3 (38.9, 39.6)	37.7 (37.7, 38.1)	<0.001
Sex (males, %)	14.1	35.6	63.7	90.5	<0.001
BMI (kg/m^2^)	25.1 (25.0, 25.2)	24.0 (23.9, 24.1)	24.0 (23.9, 24.1)	23.3 (23.2, 23.4)	<0.001
Metabolic syndromes (yes, %)	27.6	24.1	25.9	18.2	<0.001
Diabetes (yes, %)	11.6	9.85	11.4	9.50	<0.001
Hyperlipidemia (yes, %)	45.9	43.1	44.3	41.6	<0.001
ALT (U/L)	16.8 (16.4, 16.9)	17.1 (16.8, 17.5)	19.1 (18.7, 19.3)	19.5 (19.3, 19.9)	<0.001
Physical activity (Mets × hour/week)	8.85 (8.58, 9.21)	9.49 (9.12, 9.87)	9.97 (9.68, 10.38)	11.0 (10.6, 11.4)	<0.001
Total energy intake (kcal/d)	2143.1 (2121.8, 2186.4)	2230.5 (2208.3, 2253.0)	2321.6 (2275.6, 2344.9)	2440.6 (2416.3, 2465.1)	0.35
Smoking status (%)					
Smoker	6.67	14.3	23.1	33.1	<0.001
Ex-smoker	1.99	4.41	6.48	8.23	<0.001
Drinker status (%)					
Everyday	1.19	2.09	4.54	5.65	<0.001
Sometime	42.3	51.6	62.2	71.1	<0.001
Ex-drinker	9.52	9.69	9.50	9.16	0.48
Educational level (≥college graduate, %)	55.6	66.1	69.0	70.0	<0.001
Employment status (%)					
Managers	39.3	42.1	45.8	42.6	<0.001
Professionals	12.0	15.8	18.2	22.9	<0.001
Household income (>10,000 Yuan, %)	29.6	31.6	34.0	32.8	<0.001
Family history of diseases (%)					
CVD	42.1	40.0	40.3	37.4	<0.001
Hypertension	56.3	54.1	53.9	51.3	<0.01
Hyperlipidemia	9.18	9.30	9.55	8.61	0.41
Diabetes	39.7	37.4	38.0	33.2	<0.001

^a^BMI, body mass index; ALT, alanine aminotransferase; CVD, cardiovascular disease.

^b^Analysis of variance or logistic regression analysis.

^c^Geometric mean (95% confidence interval) (all such values).

**Table 2:** Adjusted Relationships of Quartiles of Grip Strength per Body Weight with NAFLD^a^.

Quartiles of grip strength per body weight (range, kg/kg)					
NAFLD	Level 1 (0.08–0.40)	Level 2 (0.40–0.48)	Level 3 (0.48–0.56)	Level 4 (0.56–1.12)	*p* for trend^b^
No. of subjects	5,240	5,239	5,238	5,240	—
No. of overall NAFLD	1,589	1,414	1,526	1,130	—
Model 1^d^	1	0.85 (0.78, 0.93)^c^	0.95 (0.87, 1.03)	0.63 (0.58, 0.69)	<0.001
Model 2^e^	1	0.91 (0.81, 1.03)	0.78 (0.68, 0.89)	0.67 (0.58, 0.78)	<0.001
Model 3^f^	1	0.89 (0.78, 1.01)	0.77 (0.67, 0.89)	0.67 (0.57, 0.79)	<0.001
NAFLD with normal ALT levels					
No. of subjects	4,871	4,874	4,865	5,015	—
No. of NAFLD with normal ALT levels	1,220	1,049	1,153	905	—
Model 1^d^	1	0.82 (0.75, 0.90)	0.93 (0.85, 1.02)	0.66 (0.60, 0.73)	<0.001
Model 2^e^	1	0.93 (0.82, 1.06)	0.80 (0.69, 0.92)	0.72 (0.62, 0.85)	<0.001
Model 3^f^	1	0.91 (0.80, 1.04)	0.79 (0.68, 0.92)	0.72 (0.61, 0.85)	<0.001
NAFLD with elevated ALT levels					
No. of subjects	4,020	4,190	4,085	4,335	—
No. of NAFLD with elevated ALT levels	369	365	373	225	—
Model 1^d^	1	0.94 (0.81, 1.10)	0.99 (0.86, 1.16)	0.54 (0.46, 0.64)	<0.001
Model 2^e^	1	0.90 (0.72, 1.13)	0.73 (0.58, 0.93)	0.55 (0.42, 0.72)	<0.001
Model 3^f^	1	0.77 (0.61, 0.98)	0.67 (0.51, 0.86)	0.53 (0.40, 0.71)	<0.01

^a^NAFLD, nonalcoholic fatty liver disease; ALT, alanine aminotransferase.

^b^Multiple logistic regression analysis.

^c^Odds ratios (95% confidence interval) (all such values).

^d^Crude.

^e^Adjusted for age, sex, and body mass index.

^f^Adjusted for age, sex, body mass index, smoking status, drinking status, physical activity, total energy intake, educational levels, employment status, household income, metabolic syndromes, diabetes, hyperlipidemia and family history of diseases including cardiovascular disease, hypertension, hyperlipidemia, and diabetes.
